# High‐Sensitivity Cardiac Troponin T Compared With Standard Troponin T Testing on Emergency Department Admission: How Much Does It Add in Everyday Clinical Practice?

**DOI:** 10.1161/JAHA.113.000204

**Published:** 2013-06-21

**Authors:** Angelika Hammerer‐Lercher, Thomas Ploner, Sabrina Neururer, Peter Schratzberger, Andrea Griesmacher, Otmar Pachinger, Johannes Mair

**Affiliations:** 1 Central Institute for Medical and Chemical Laboratory Diagnostics, Innsbruck Medical University, Innsbruck, Austria (A.H.L., A.G.); 2 Department of Internal Medicine, Innsbruck Medical University, Innsbruck, Austria (T.P., P.S., O.P., J.M.); 3 Department of Medical Statistics, Informatics and Health Economics, Innsbruck Medical University, Innsbruck, Austria (S.N.)

**Keywords:** cardiac troponin T, diagnosis, emergency department, high sensitivity, myocardial infarction, risk stratification

## Abstract

**Background:**

We compared high‐sensitivity cardiac troponin T (hs‐cTnT) and standard cTnT for acute myocardial infarction (AMI) diagnosis in everyday clinical practice of an emergency department (ED).

**Methods and Results:**

cTnT was measured in 2384 consecutive patients (60±21 years, 52% female) on ED admission. Readmissions to the ED (n=720) and mortality (n=101) were followed for an average period of 239±49 days. There were 53 AMIs (delay, 1 to 96 hours; median, 3 hours), 440 chest pain patients, 286 dyspnea patients, 785 acute or chronic cardiac diseases, and 540 neurological diseases, with the remaining having various internal diseases. The diagnostic performances of hs‐ and standard cTnT were comparable for AMI diagnosis (area under receiver operating characteristics curves [ROC AUC], 0.91±0.02 versus 0.90±0.03; *P*=0.31). Using the 99th‐percentile cutoff, the sensitivities and specificities for AMI in the whole population were 91% and 74% for hs‐cTnT and 89% and 80% for standard cTnT. hs‐cTnT detected significantly more patients with cardiac diseases (ROC AUC, 0.77±0.01 versus 0.67±0.01; *P*<0.001). hs‐cTnT and standard cTnT were significant predictors of ED readmissions but not of mortality, but both were not independent predictors of ED readmissions or the combined end point of readmission or mortality in binary logistic regression analysis.

**Conclusions:**

In unselected ED patients the diagnostic performances of hs‐cTnT and standard cTnT for AMI diagnosis did not differ significantly. hs‐cTnT detected significantly more cardiac diseases. hs‐cTnT and standard cTnT were not independent predictors of ED readmissions and mortality from all causes.

## Introduction

During the past 2 decades cardiac troponin (cTn) has emerged as the criterion biomarker for the diagnosis of acute myocardial infarction (AMI), and during recent years the analytical sensitivity and assay precision at the low measuring range of cTn assays has been continuously improved to fulfill the analytical criteria of current guidelines,^1–2^ that is, a total coefficient of variation (CV) of <10% at the 99th percentile of troponin concentrations of a healthy reference population (=the recommended upper reference limit [URL]). Recently, the first assay that fulfills these criteria in routine clinical laboratories has been introduced for routine cTn testing outside the United States, the so‐called high‐sensitivity cardiac troponin T (hs‐cTnT) assay.^3^ hs‐cTn assays permit precise measurement of cTn concentrations in a significant number of apparently illness‐free individuals and thus more precise calculation of the 99th‐percentile cTn concentration in reference subjects.^3–5^ These assays measure cTn in the single‐digit range of nanograms per liter, with some research assays even below 1 ng/L.^3,5–6^ It has been postulated that hs‐cTn assays will improve early AMI diagnosis, and the first clinical studies in preselected and highly preselected chest pain patient populations indeed demonstrated a significant improvement in early diagnostic sensitivity for AMI.^7–9^ However, the clinical benefits of hs‐cTn testing in everyday clinical practice are still a matter of debate, and, therefore, we investigated this issue in a large consecutive emergency department (ED) population in which cTnT testing was requested liberally by the attending physician by parallel measurement of standard cTnT (fourth‐generation cTnT assay) with the new hs‐cTnT assay.

## Methods

We compared the diagnostic performances of the hs‐cTnT assay with its previous fourth generation (=standard) cTnT assay generation (Roche Diagnostics, Vienna, Austria) during a period of ≈2 months (March 6 until May 2, 2010) in everyday clinical practice in an ED treating mainly adults with medical or neurological emergencies to evaluate the potential clinical benefits for routine diagnosis. The University Hospital of Innsbruck is a tertiary‐care center, but at the same time it is the only hospital in Innsbruck (a city with ≈140 000 inhabitants), and, therefore, it is also a primary care center for Innsbruck and the surrounding villages. Thus, it is the only hospital for readmissions to the ED in this area. The emergency care for adults at our hospital is mainly organized in 3 emergency departments: 1 for trauma, 1 for obvious surgical emergencies, and 1 treating nonsurgical, mainly internal and neurological emergencies. This study was carried out in the latter ED, and emergencies were first seen by internists or neurologists according to their key symptoms. This study was approved by the local ethical committee.

During the study period a total of 5946 patients were seen in this ED, 4476 primarily by internists and 1470 by neurologists (2795 males, 3151 females; mean age, 50 years; range, 17 to 101 years). cTnT was measured only on request of the attending physicians in 2384 patients (60±21 years, 52% female) on ED admission. It was only ordered in patients treated by internists or neurologists.

Patients were classified according to hospital discharge diagnoses, and AMI diagnosis was based on the universal definition of AMI criteria published in 2007.^1^ However, all discharge diagnosis of AMI, acute coronary syndrome, and unstable angina were reevaluated by a cardiologist (J.M.) on the basis of the available stored data of hospital charts using serial fourth‐generation cTnT values with a cutoff value of 10 ng/L, with a rise and/or fall as the biochemical criterion of acute myocardial injury according to the universal definition of AMI.^1^

Baseline characteristics of the whole study population with ordered cTnT, chest pain patients, and non–chest pain patients are shown in Tables 1 and 2. Seven hundred eighty‐five patients had acute or chronic cardiac diseases including 53 AMI patients (delay from onset to admission, 1 to 96 hours; median, 3 hours; in 16 patients the exact delay was not known) comprising 30 ST‐segment‐elevation AMIs (STEMIs) and 23 non‐STEMIs. Five hundred forty patients had neurological diseases including 88 patients with stroke (60 ischemic stroke and 28 intracranial bleedings). The remaining 875 patients suffered from various internal diseases. Four hundred forty patients presented with the key symptom chest pain (110 patients had typical angina pectoris symptoms; see Table 2) and 286 patients with acute dyspnea.

**Table 1.** Baseline Characteristics of the Study Population Subdivided by the Key Symptom Chest Pain

	Whole Study Population	Chest Pain Patients	Non–Chest Pain Patients	Chest Pain vs Non–Chest Pain Patients (*P* Value)
Number of patients	2384	440	1944	
Female	1245 (52.6%)	210 (47.7%)	1034 (53.2%)	0.037
Age (y)	60±20.9	55.7±19.8	60.9±21	<0.001
Cardiovascular history				
Hypertension	1100 (46.1%)	204 (46.4%)	896 (46.1%)	0.92
Diabetes	276 (11.6%)	33 (7.5%)	243 (12.5%)	0.003
Known CAD	397 (16.3%)	84 (19.1%)	313 (16.1%)	0.13
Known heart failure	173 (7.3%)	17 (3.9%)	156 (8.0%)	0.002
Creatinine (mg/dL), median (interquartile range)	0.86 (0.72 to 1.04)	0.87 (0.74 to 1.01)	0.86 (0.72 to 1.05)	0.79
Diagnosis				
AMI	53 (2.2%)	40 (9.1%)	13 (0.7%)	<0.001
Cardiac disease	787 (33%)	181 (41.1%)	606 (31.2%)	<0.001
Pulmonary disease	301 (12.6%)	42 (9.5%)	259 (13.3%)	0.031
Cerebrovascular disease	133 (5.6%)	1 (0.2%)	132 (6.8%)	<0.001
Other diseases	1540 (64.6%)	261 (59.3%)	1279 (65.8%)	0.010
Admission ECG				
ST elevation	42 (1.8%)	32 (7.3%)	10 (0.5%)	<0.001
ST depression	50 (2.1%)	17 (3.9%)	33 (1.7%)	0.004
Other pathological signs	596 (25%)	92 (20.9%)	504 (25.9%)	0.028
Normal ECG	1403 (58.9%)	295 (67%)	1108 (57%)	<0.001
Admission troponin				
Fourth‐generation cTnT (75% percentile)	<10 ng/L	<10 ng/L	<10 ng/L	0.32
hs‐cTnT (ng/L), median (interquartile range)	5 (<5 to 16.4)	5 (<5 to 14.8)	5 (<5 to 16.9)	0.024

CAD indicates coronary artery disease; AMI, acute myocardial infarction; cTnT, cardiac troponin T; hs, high sensitivity.

**Table 2.** Baseline Characteristics of Chest Pain Patients Subdivided by Discharge Diagnosis

	Chest‐Pain Patients		
	AMI	Other Cardiac Diseases	Other Diseases
Number of patients	40	141	259
Female	13 (32.5%)	62 (44%)	135 (51.7%)
Age (y)	65±13.3	72±16	45±17.4
Delay (h), median (interquartile range)	2 (1 to 8)	3 (2 to 8)	3 (2 to 10)
Cardiovascular history			
Hypertension	25 (62.5%)*†	113 (80.1%)‡	66 (25%)
Diabetes	2 (5%)	23 (16,3%)‡	8 (3.1%)
Known CAD	10 (25%)*†	73 (51.8%)‡	1 (0.4%)
Known heart failure	2 (5%)†	15 (10.6%)‡	0 (0%)
Renal function			
Creatinine (mg/dL), median (interquartile range)	0.95† (0.79 to 1.10)	0.92‡ (0.81 to 1.15)	0.83 (0.71 to 0.96)
Admission ECG			
ST elevation	26 (65%)*†	5 (3.5%)‡	3 (1.1%)
ST depression	3 (7.5%)	6 (4.3%)	8 (3.1%)
Other pathological signs	7 (17.5%)*	58 (41.1%)‡	27 (10.3%)
Normal ECG	4 (10%)*†	71 (50.4%)‡	220 (84.3%)
Admission troponin			
Fourth‐generation cTnT (ng/L), median (interquartile range)	62*† (27.5 to 410.8)	<10 (<10 to 11)	<10 (=75% percentile)
hs‐cTnT (ng/L), median (interquartile range)	81.7L*† (36.8 to 428)	10.5‡ (<5 to 20.6)	<5 (=75% percentile)

AMI indicates acute myocardial infarction; CAD, coronary artery disease; cTnT, cardiac troponin T; hs, high sensitivity.

Significant differences between groups after Bonferroni correction:

* AMI compared with other cardiac diseases;

^†^ AMI compared with other diseases;

^‡^ cardiac diseases compared with other diseases.

ED readmissions and mortality were recorded for a follow‐up period of up to 274 days (mean, 239±49 days; range, 1 to 274 days**)**, and a total of 720 patients were readmitted after 1 to 270 days (mean, 76 days) including 187 patients who were readmitted because of cardiac diseases on average after 33 days (range, 1 to 163 days). One hundred one patients died during follow‐up after 1 to 274 days (mean, 230 days).

hs‐cTnT and standard (fourth‐generation) cTnT were measured by assays from Roche Diagnostics (Vienna, Austria) as previously described.^3,10^ The lot numbers of hs‐cTnT assays were 15340101 and 15712001. The analytical limit of detection (LoD) and the 99th‐percentile URL were both 10 ng/L (=0.01 μg/L), and the 10% coefficient of variation (CV) cutoff value was 30 ng/L (=0.03 μg/L) for the fourth‐generation cTnT assay and 5 ng/L (LoD), 14 ng/L (URL), and 13 ng/L (10% CV cutoff) for the hs‐cTnT assay.^3,10^ Recently, the sex‐specific 99th percentiles for the hs‐cTnT have been published ^11^ which are 20 ng/L for males and 13 ng/L for females. In our routine hospital laboratory the following interassay CV values were found: fourth‐generation cTnT assay, 3.4% at a concentration of 64 ng/L and 1.9% at 2430 ng/L (n≥25); hs‐cTnT, 1.1% (n=23) and 1.0% (n=30) at concentrations of 27.7 and 2190 ng/L, respectively.

During this assay transition period the results of both assays were reported to ED physicians in micrograms per liter, but physicians were instructed to base their clinical decisions only on the standard cTnT assay results using the 99th percentile (=10 ng/L) as a decision limit.^1^

### Statistics

All statistical analysis was performed using either SPSS 20.0 (International Business Machines Corporation, Armonk, NY) or MedCalc 11.3 (Med Calc Software bvba, Mariakerke, Belgium) statistical software packages. Continuous data are given as mean and standard deviation or median and interquartile range (IQR) and dichotomous variables as percentages. The Kruskal–Wallis test, the Mann–Whitney *U* test, and the chi‐square test were used for group comparisons. For assessing diagnostic performances, we performed receiver operating characteristics (ROC) curve analysis. For the statistical comparison of ROC curves, the method of De Long was used. ROC analysis was also used to calculate medical decision limits for hs‐cTnT, which yielded a negative likelihood ratio (LR) of about 0.1 and a positive LR >10 for AMI. The prognostic performance of variables for prediction of ED readmission was assessed by a Kaplan–Meier survival curve and binary logistic regression analysis. For binary logistic regression analysis, standard (fourth‐generation) cTnT and hs‐cTnT (both non‐normally distributed) were log‐transformed, and because of the very high correlation of both variables, the analysis was performed separately using either hs‐cTnT or standard cTnT. Bland–Altman, Passing–Bablok, and Spearman correlation coefficient were calculated to compare the assay agreement of the 2 cTnT tests. Samples with values below the LoD or higher than the dilution limit in 1 assay were excluded for the correlation calculation. All statistical testing was done 2‐sided, and *P* values <0.05 were considered statistically significant.

## Results

### Correlations Between the hs‐cTnT and the Fourth‐Generation cTnT Assays and Agreement in Patient Classification as cTnT Positive or Negative

**Figure 1.**
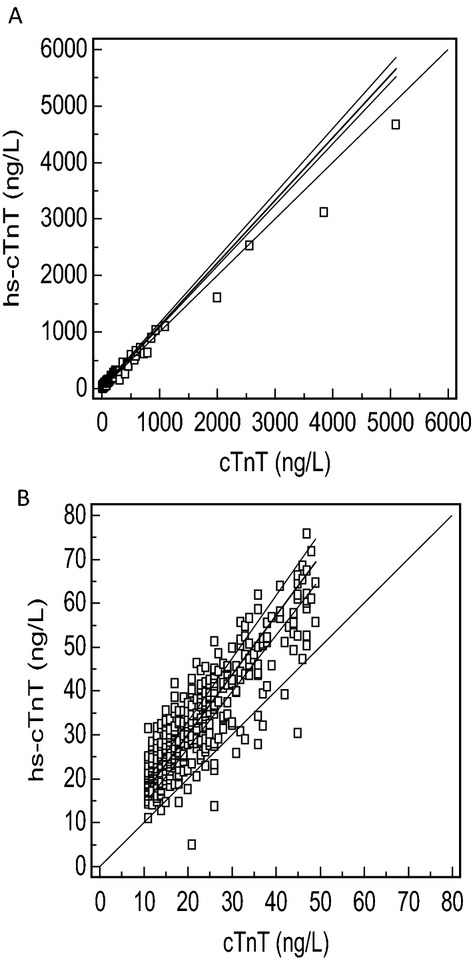
Passing and Bablok regression analysis for the analytical comparison of the hs‐cTnT and standard cTnT (fourth‐generation) assays. A, There was a good agreement between both assays in the whole study group, with cTnT values above the lower limit of detection and below the dilution limit in both assays (hs‐cTnT=7.59+1.11×cTnT [fourth generation]; n=506). B, The agreement of both methods was weaker when values in the lower measuring range (>10 and <50 ng/L) of the fourth‐generation cTnT assay were compared (hs‐cTnT=3.05+1.36×cTnT (fourth generation); n=376). hs‐cTnT indicates high‐sensitivity cardiac troponin T.

Overall, both assays correlated closely (*r*=0.93, *P*<0.0001, n=506). If only samples in the lower measuring range (>10 and <50 ng/L) of the fourth‐generation cTnT assay were compared between both assays, the correlation was weaker (*r*=0.835, *P*<0.0001, n=376), with ≈38% higher hs‐cTnT values with a mean absolute bias of 10 ng/L (see Figure 1). Using the URLs for both assays as decision limits (14 ng/L for hs‐cTnT and 10 ng/L for standard cTnT), the overall agreement of both assays in the classification of patients as cTnT positive and negative was 94%. One hundred thirty‐eight patients (5.8%) were hs‐cTnT positive but standard cTnT negative including 5 patients with unstable angina and 24 patients with other cardiac diseases.

### Comparison of Diagnostic Performances of Both cTnT Assays for AMI Diagnosis

#### Chest pain patients

The difference of areas under ROC curve (AUC) for AMI diagnosis was small, and AUCs (hs‐cTnT, 0.94 [95% CI, 0.91 to 0.96]; fourth‐generation cTnT, 0.91 [95% CI, 0.88 to 0.94]) did not differ significantly (*P*=0.08). Sensitivities, specificities, predictive values (AMI prevalence, 9.1%), and likelihood ratio of both cTnT assays are listed in Table 3. The hs‐cTnT ROC criterion cutoff optimizing sensitivity and specificity in this chest pain population was 20 ng/L. Undetectable hs‐cTnT (<5 ng/L) ruled out AMI with very high probability, and on the other hand a hs‐cTnT admission concentration >30 ng/L ruled in AMI with very high probability (see Table 3).

**Table 3.** Comparison of the Diagnostic Performances of High‐Sensitivity and Standard (Fourth‐Generation) Cardiac Troponin T for Acute Myocardial Infarction Diagnosis in 440 Chest Pain Patients

Assay	Cutoff (ng/L)	Sensitivity (%)	Specificity (%)	+PV (%)	−PV (%)	+LR	−LR
hs‐cTnT	5 (LoD)	98 (87 to 99.9)	65 (60 to 70)	22 (16 to 28)	99.6 (98 to 100)	2.8 (2.5 to 3.0)	0.04 (0.01 to 0.3)
hs‐cTnT	14 (URL)	90 (76 to 97)	80 (76 to 84)	31 (23 to 41)	99 (97 to 99.7)	4.6 (4.1 to 5.1)	0.12 (0.05 to 0.3)
hs‐cTnT	20 (ROC criterion value)	88 (73 to 96)	88 (84 to 91)	42 (31 to 53)	99 (97 to 99.5)	7.1 (6.3 to 8.1)	0.14 (0.06 to 0.3)
hs‐cTnT	30 (cutoff optimized for +LR)	80 (64 to 91)	93 (90 to 96)	54 (41 to 67)	98 (96 to 99)	11.9 (10.1 to 13.9)	0.21 (0.10 to 0.40)
Fourth gen. cTnT	10 (URL, ROC criterion value)	88 (73 to 96)	88 (84 to 91)	42 (31 to 54)	99 (97 to 99.5)	7.3 (6.5 to 8.2)	0.14 (0.06 to 0.3)
Fourth gen. cTnT	30 (10% CV)	75 (59 to 87)	97 (95 to 98)	71 (55 to 84)	98 (95 to 99)	25 (20.9 to 29.91)	0.26 (0.1 to 0.6)

AMI prevalence 9.1%; 95% CI is given in parentheses. PV indicates predictive value; LR, likelihood ratio; hs, high sensitivity; cTnT, cardiac troponin T; LoD, lower limit of detection; URL, upper reference limit 99th percentile; ROC, receiver operating characteristics; CV, coefficient of variation.

Similar results were found in the subgroups of patients presenting with dyspnea (hs‐cTnT, 0.86 [95% CI, 0.82 to 0.90]; versus standard cTnT, 0.89 [95% CI, 0.85 to 0.92]; *P*=0.32, n=286) or patients with chest pain or dyspnea (hs‐cTnT, 0.90 [95% CI, 0.87 to 0.92]; versus standard cTnT, 0.88 [95% CI, 0.86 to 0.90]; *P*=0.16, n=726).

#### Whole study population

**Figure 2.**
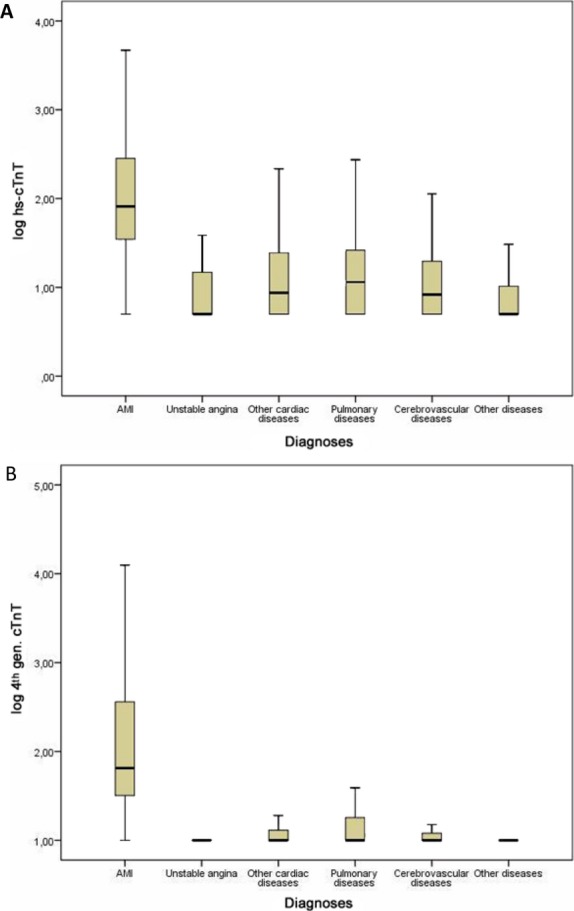
hs‐cTnT (A) and standard cTnT concentration distributions (B) in the different disease categories. Data given as box plots. hs‐cTnT and fourth‐generation cTnT concentrations of AMI patients were significantly higher compared with cTnT concentrations of all other disease groups (Kruskal–Wallis test, *P*<0.001). log fourth gen. cTnT indicates logarithmical fourth‐generation cardiac troponin T concentrations; log hs‐cTnT, logarithmical high‐sensitivity cardiac troponin T concentrations; AMI, acute myocardial infarction.

**Figure 3.**
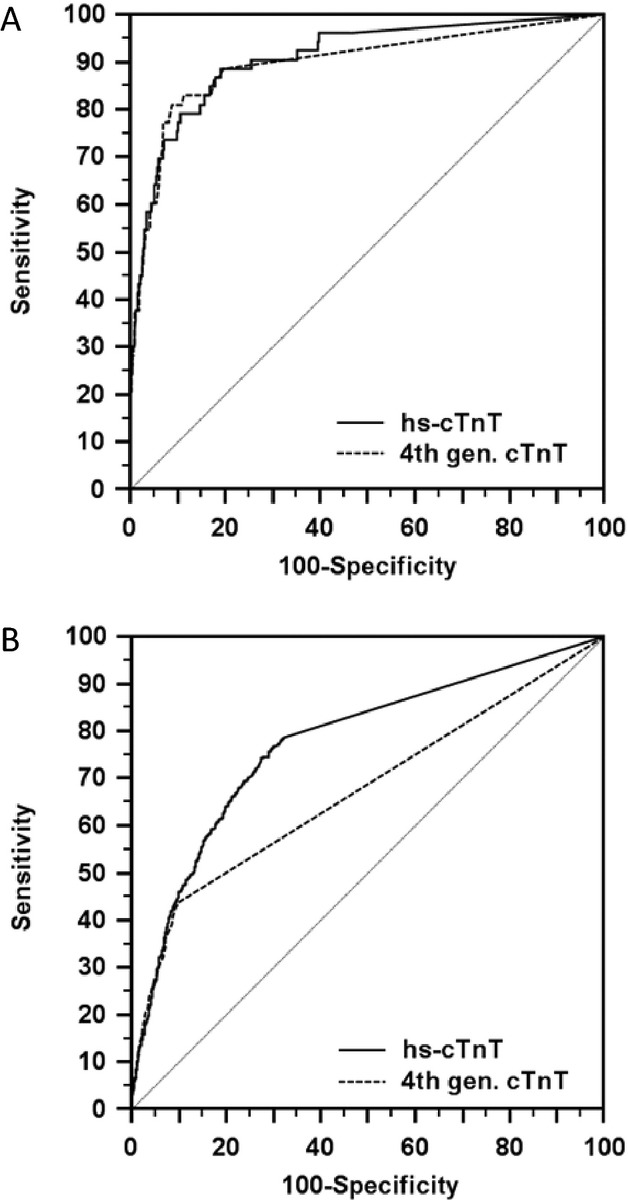
ROC analysis of high‐sensitivity and standard cardiac troponin T for the detection of acute myocardial infarction (A) and cardiac diseases (B) in the whole study population. There were no significant differences between both assays for AMI detection (A), but hs‐cTnT detected significantly more patients with any cardiac diseases (B). ROC indicates receiver operating characteristics; AMI, acute myocardial infarction; hs‐cTnT, high‐sensitivity cardiac troponin T.

hs‐cTnT and fourth‐generation cTnT concentrations of different disease groups are shown in Figure 2. The overall diagnostic performances for AMI diagnosis of both assay generations were comparable (ROC AUC, 0.91±0.02 versus 0.90±0.03; *P*=0.31; see Figure 3A). Diagnostic performance characteristics of various cutoff limits are listed in Table 4. The hs‐cTnT URL (14 ng/L) ruled out AMI with high probability but acceptable positive predictive value, and the decision limit 45 ng/L ruled in AMI with high probability (see Table 4).

**Table 4.** Comparison of the Diagnostic Performances of High‐Sensitivity and Standard (Fourth generation) Cardiac Troponin T for Acute Myocardial Infarction Diagnosis in the Whole Study Population (n=2384)

Assay	Cut‐off (ng/L)	Sensitivity (%)	Specificity (%)	+PV (%)	−PV (%)	+LR	−LR
hs‐cTnT	5 (LoD)	96 (87 to 100)	54 (51 to 56)	5 (3 to 6)	99.8 (99.4 to 100	2.1 (1.9 to 2.2)	0.07 (0.02 to 0.30)
hs‐cTnT	14 (URL)	91 (79 to 97)	74 (72 to 76)	7 (5 to 10)	99.7 (99.3 to 99.7)	3.4 (3.1 to 3.8)	0.13 (0.06 to 0.30)
hs‐cTnT	20 (ROC criterion value)	89 (77 to 96)	81 (79 to 82)	10 (7 to 12)	99.7 (99.3 to 99.9)	4.6 (4.2 to 5.1)	0.14 (0.07 to 0.30)
hs‐cTnT	45 (cut‐off optimized for +LR)	70 (56 to 82)	93 (92 to 94)	19 (14 to 25)	99.3 (98.8 to 99.6)	10.3 (8.6 to 12.3)	0.32 (0.20 to 0.50)
Fourth gen. cTnT	10 (URL)	89 (77 to 962)	80 (79 to 82)	9 (7 to 12	99.7 (99.3 to 99.9)	4.5 (4.1 to 5.0)	0.14 (0.07 to 0.3)
Fourth gen. cTnT	30 (10% CV)	73 (60 to 84)	93 (92 to 94)	20 (15 to 26)	99.3 (98.9 to 99.6)	10.6 (9.1 to 12.5)	0.3 (0.2 to 0.5)
Fourth gen. cTnT	25 (ROC criterion value)	81 (68 to 91)	91 (90 to 92)	17 (13 to 23)	99.5 (99.1 to 99.8)	9.2 (8.1 to 10.5)	0.21 (0.1 to 0.4)

95% CI is given in parenthesis. PV indicates predictive value; LR, likelihood ratio; hs, high‐sensitivity; cTnT, cardiac troponin T; LoD, lower limit of detection; URL, upper reference limit 99th%; ROC, receiver operating characteristics; gen, generation; CV, coefficient of variation.

#### Subgroup analysis

##### Early sensitivities of troponin assays within 3 hours from chest pain onset

Subgroup analysis in patients presenting within ≤3 hours from symptom onset (ROC AUC, 0.85±0.05 versus 0.84±0.05; *P*=0.61, n=156) and patients presenting thereafter (ROC AUC, 0.95±0.02 versus 0.96±0.02; *P*=0.48, n=135) did not reveal different results in the comparison of assays, but a worse overall diagnostic performance of cTnT in patients presenting early after symptom onset. The early sensitivities were similar for both assays if the 99th‐percentile URLs were used as medical decision limits for both assays (83% [95% CI, 63% to 95%] versus 79% [95% CI, 58% to 93%]). Only when the 10% CV limit (30 ng/L) was used as a medical decision limit for the fourth‐generation cTnT assay did the early sensitivity of hs‐cTnT tend to be nonsignificantly higher (83% [95% CI, 63% to 95%] versus 67% [95% CI, 45% to 85%]).

##### Influence of sex

The influence of sex on optimal decision limits according to ROC analysis was tested in the whole study population as well as in the subgroup of chest pain patients (see Table 5). In the whole male study population the optimal decision limit for AMI was 20 ng/L, which is identical to the sex‐specific 99th‐percentile URL published by Apple et al^11^ for males; in the subgroup of male chest pain patients it was markedly higher (40 ng/L). All female groups showed higher ROC criterion values for AMI diagnosis than the sex‐specific URL (=13 ng/L) published by Apple et al.^11^

**Table 5.** Diagnostic Performances of Cardiac Troponin T by Sex or Renal Function in the Whole Study Population and the Subgroup of Chest Pain Patients

Group	Creatinine	Troponin Assay	ROC Criterion Cutoff (ng/L)	ROC AUC
All male patients	—	hs	20	0.88 (0.86 to 0.90)
All male patients	—	Standard	25	0.86 (0.84 to 0.88)
All female patients	—	hs	33	0.93 (0.92 to 0.95)
All female patients	—	Standard	21	0.94 (0.93 to 0.95)
All patients	>0.8	hs	20	0.86 (0.84 to 0.88)
All patients	>0.8	Standard	25	0.86 (0.83 to 0.88)
All patients	≤0.8	hs	33	0.94 (0.93 to 0.96)
All patients	≤0.8	Standard	12	0.94 (0.92 to 0.95)
Male chest pain patients	—	hs	40	0.92 (0.88 to 0.95)
Male chest pain patients	—	Standard	31	0.88 (0.83 to 0.92)
Female chest pain patients	—	hs	21	0.97 (0.94 to 0.99)
Female chest pain patients	—	Standard	11	0.97 (0.94 to 0.99)
Chest pain patients	>0.8	hs	42	0.89 (0.84 to 0.93)
Chest pain patients	>0.8	Standard	25	0.87 (0.82 to 0.91)
Chest pain patients	≤0.8	hs	21.1	0.98 (0.95 to 0.99)
Chest pain patients	≤0.8	Standard	11	0.96 (0.92 to 0.98)

Patients were grouped using the median of creatinine concentrations (0.8 mg/dL); 95% confidence intervals are given in parentheses. The diagnostic performances of troponin assays were worse in patients with creatinine >0.8 mg/dL. ROC AUC indicates area under receiver operating characteristics curve; hs, high sensitivity.

##### Influence of renal function

To evaluate the influence of renal function, patients were grouped according to the median of creatinine concentrations (=0.8 mg/dL; see Table 5). There were no significant differences in the diagnostic performances of both assays in these subgroups in chest pain patients as well as in the whole study group. In chest pain patients the optimal decision limits for AMI diagnosis according to ROC analysis were markedly higher (hs‐cTnT, 42 ng/L) in patients with creatinine concentrations >0.8 mg/dL. The delay from onset of symptoms to admission did not differ significantly (*P*>0.65) between both creatinine groups in chest pain patients as well as AMI patients. In addition, the frequency of ST‐segment‐elevation AMIs did not differ significantly between creatinine groups either (*P*=0.78).

### Comparison of Diagnostic Performances of Both Assays for Detection of Any Acute or Chronic Cardiac Disease

hs‐cTnT detected significantly more patients with any acute or chronic cardiac diseases (ROC AUC, 0.77±0.01 versus 0.67±0.01; *P*<0.001; see Figure 3B) when compared with standard cTnT. An undetectable hs‐cTnT excluded cardiac diseases with a high negative predictive value (87%) and a negative LR of 0.31.

Similar results were found in the subgroups of patients presenting with chest pain, dyspnea, or patients with chest discomfort or dyspnea (data not shown).

### Prediction of Emergency Department Readmissions or Mortality

hs‐cTnT and standard cTnT were significant predictors of ED readmissions of any cause and of cardiac causes in Kaplan–Meier survival analysis (see Table 6). The odds ratios for hs‐cTnT‐positive/standard cTnT‐negative and hs‐cTnT‐positive/standard cTnT‐positive patients for the prediction of cardiac and all‐cause readmissions did not differ significantly (*P*>0.23). hs‐cTnT and standard cTnT did not significantly predict mortality in the whole population as well as in the subgroup of chest pain patients. Similarly, they were not independent predictors of ED readmissions, cardiac‐related ED readmissions, or the combined end point of mortality or cardiac‐related ED readmissions in binary logistic regression analysis.

**Table 6.** Univariable Risk Prediction of Emergency Department Readmission During Follow‐Up

Group	Cause of Readmission	Odds Ratio
hs‐cTnT pos/standard cTnT pos	Any	2.1 (1.7 to 2.6)
hs‐cTnT pos/standard cTnT neg	Any	2.4 (1.7 to 3.5)
hs‐cTnT pos/standard cTnT pos	Cardiac	2.2 (1.6 to 3.0)
hs‐cTnT pos/standard cTnT neg	Cardiac	1.4 (0.76 to 2.6)

Odds ratio for the comparison with troponin‐negative patients in both assays are listed. The 99th‐percentile upper reference limits were used for both troponin assays for the discrimination positive and negative. The odds ratios of hs‐cTnT‐positive/standard cTnT‐negative and hs‐cTnT‐positive/standard cTnT‐positive patients did not differ significantly (*P*>0.23). The 95% confidence intervals are given in parentheses. hs indicates high‐sensitivity; cTnT, cardiac troponin T; pos, positive; neg, negative.

## Discussion

The increasing use of hs‐cTn assays in everyday clinical practice in Europe has led to considerable confusion and uncertainties on their use and usefulness in everyday clinical practice. This resulted in an increasing number of published expert opinions on how to use hs‐cTn and on the interpretation of its test results.^12–14^ However, these recommendations until now have not been based on solid large‐scale comparative study data from everyday clinical use of hs‐cTn, and thus the present study is the first large comparative study on hs‐cTnT and standard cTnT in about 2400 consecutive ED patients based on everyday liberal clinical ordering of cTnT testing on request of the attending physician in the ED without a restrictive study protocol, which usually leads to preselection of high‐risk patients.

The key findings of our study are: (1) the diagnostic performances of standard and hs‐cTnT for AMI diagnosis were comparable on ED admission in everyday clinical use in unselected patients; (2) the early sensitivities for AMI diagnosis of both assays including subgroup analysis of patients admitted within 3 hours from chest pain onset did not differ significantly, and the negative predictive values were comparable as well if for both assays' 99th‐percentile URLs were used as medical decision limits (14 ng/L for hs‐cTnT and 10 ng/L for standard cTnT); (3) the positive predictive value for AMI of hs‐cTnT was lower in our low‐prevalence population compared with the standard assay; (4) hs‐cTnT detected signicantly more patients with cardiac diseases, which could not be outweighed by using the 99th‐percentile URL as a decision limit for the standard cTnT assay; and (5) hs‐cTnT was not an independent predictor of ED readmissions and mortality from all causes in binary logistic regression analysis.

Our finding of comparable diagnostic performances of both cTnT assays for AMI diagnosis in a low‐probability population on ED admission appears to be in contrast to some previous reports.^7–9^ However, in many studies in this field clinically more preselected ED or highly preselected chest pain unit populations were investigated,^7–9,7–16^ and also different medical decision limits for standard and hs‐cTn assays were used, that is, a 10% CV limit for the standard cTn assay and the 99th‐percentile URL for the hs‐cTn assay. Thereby, possible differences between assays are overstated. Only when using the 10% CV value (30 ng/L) as a medical decision limit for the standard cTnT assay did we find a higher early diagnostic sensitivity for the hs‐cTnT assay in patients admitted within 3 hours from chest pain patients (83% versus 67%). However, this approach is not guideline driven,^1^ and when we used the 99th‐percentile URL for both assays, the early sensitivities were comparable (91% versus 89%) on ED admission. This has been postulated previously,^17^ but this is the first large study that proves this hypothesis.

Our subgroup of 440 chest pain patients had an AMI prevalence of about 10%, which reflects everyday clinical conditions of a low‐risk population presenting to the ED. In this subgroup, standard and hs‐cTnT diagnostic performances did not differ significantly either. This is in agreement with a smaller recently published multicenter study in 317 chest pain patients with an AMI prevalence of 14% in which the ROC AUCs of hs‐cTnT and standard cTnI for AMI diagnosis did not differ significantly either.^18^ A drawback of this study was that in the different centers different cardiac troponin I assays with differences in the assay precision at the low measuring range were used for comparison. However, we can confirm these results with our study results comparing the hs‐ and standard cTnT assay performances. Reichlin et al,^7^ by contrast, reported a statistically significant difference between hs‐cTnT and standard cTnT (ROC AUC, 0.90 versus 0.96) in a more preselected (AMI prevalence, 17%) but larger chest pain patient group (n=718) with higher statistical power than our study. However, the absolute difference of the AUC in both assays (0.06) reported by Reichlin et al was small as well.

We confirm that despite its excellent negative predictive value (>99%) and a negative likelihood ratio close to 0.1, a single hs‐cTnT concentration <14 ng/L on ED admission would not allow ruling out AMI with 100% certainty, and thus we cannot avoid repeat hs‐cTnT testing several hours later. On the other hand, in the whole study population, an hs‐cTnT admission concentration >45 ng/L revealed a high positive LR for AMI (>10) with a still‐acceptable negative LR (≈0.3). In the subgroup of chest pain patients, admission hs‐cTnT values <5 ng/L ruled out AMI with an excellent negative LR (0.04), and values >30 ng/L predicted AMI with a high positive LR (≈12).

Most of the benefits, that is, increased early sensitivity for AMI and increased sensitivity for small myocardial infarctions of hs‐cTnT assays for routine AMI diagnosis can be achieved by the recommended application of the 99th‐percentile URL as a medical decision limit with the standard cTnT assay,^1^ however, at the price of reduced specificity for AMI, which was also seen with the use of hs‐cTnT in everyday practice. This is clearly demonstrated by our results, and this problem has been underestimated in previously published studies,^7–9^ in particular those investigating highly clinically preselected chest pain unit patient populations instead of low probability ED populations with complex comorbidities as we did in our study. Consequently, in the whole study population, the ROC criterion value hs‐cTnT cutoff (20 ng/L) balancing sensitivity and specificity for AMI diagnosis was higher than the generally used URL. Our sex‐specific analysis supports the clinical use of hs‐cTnT sex‐specific 99th‐percentile URLs published by Apple et al.^11^ In chest pain patients, however, we found higher AMI decision limits for both males and females.

cTn has never been a specific marker of acute coronary syndromes, but with the use of hs‐cTn assays this becomes much more evident clinically. Instead, it has always been a marker of myocardial injury of any cause. In our study significantly more patients with cardiac diseases were detected with the hs‐cTnT assay, which could not be outweighed by using the 99th‐percentile URL for the standard cTnT assay as a medical decision limit. This observation is explained by the better assay precision at the low measuring range that allows a more accurate detection of small amounts of myocardial injury. Our results show that increased awareness of alternative causes of acute and chronic low‐grade myocardial injury with consecutive cTn release is necessary given the high prevalence of hs‐cTnT concentrations greater the URL in the different disease groups.

Data on the predictive value of cTnT for ED readmissions are limited. cTnT admission concentrations (measured with both assays) predicted ED readmissions (cardiac related as well as total) during follow‐up in univariable analysis, and hs‐cTnT concentrations detected additional patients with about a 2‐fold higher risk of ED readmissions, which was comparable to the risk of patients with positive hs‐cTnT and standard cTnT concentrations. However, in binary logistic regression analysis neither hs‐cTnT nor standard cTnT independently predicted ED readmissions or the combined end‐point mortality or ED readmissions during follow‐up. A strength of this analysis is that our University Hospital is the only hospital in the city, and readmissions to other hospitals are not a matter of concern. In contrast to previous reports in clinically preselected high‐risk ED populations,^16,19^ we did not find a predictive value of cTnT for mortality in less preselected ED patients.

The following limitations of the current study merit consideration. This observational study was not blinded, as the intention was that ED physicians get used to hs‐cTnT test results during the transition phase. Although ED physicians were instructed just to use the standard cTnT test results for their clinical decisions, we cannot rule out that occasionally their decisions were influenced by seeing the hs‐cTnT test results. The reasons why neurologists ordered cTnT so frequently in patients presenting with key neurological symptoms could not be sufficiently elucidated retrospectively. The study was based on a single cTnT measurement on ED admission, and we could not evaluate cTnT kinetics because the number of patients with subsequent serial hs‐cTnT data in the ED was too small for meaningful kinetic analysis. Because of incomplete serial hs‐cTnT testing when patients were transferred from the ED, in addition, reclassification of all patients using hs‐cTnT kinetic analysis was not possible. The diagnosis of AMI was based on the universal definition of AMI ^1^ using standard cTnT as the biomarker of myocardial damage using the 99th‐percentile cutoff value. However, 5 patients classified as having unstable angina had hs‐cTnT concentrations >14 ng/L on ED admission and would be classified as non‐STEMI using hs‐cTnT as the criterion biomarker in case of significant hs‐cTnT changes in serial testing. Thus, it can be expected that some patients with unstable angina would switch to the non‐STEMI category with the implementation of routine hs‐cTnT testing. Finally, it must also be noted that the hs‐cTnT assay has not yet received clearance and is still not available for routine use in the United States.

In conclusion, the hs‐cTnT assay provides improvements as well as challenges, and the optimal balance between sensitivity and specificity is provided by listing different cutoff values in our tables that are either balanced for sensitivity and specificity (ROC criterion values) or emphasize either positive or negative likelihood ratios as clinically needed. Elevated hs‐cTnT concentrations are common in non‐AMI patients resulting in a challenging differential diagnosis. Thus, particularly in non–chest pain patients, hs‐cTnT does not necessarily improve AMI diagnosis in the ED despite slightly higher early sensitivity. As with almost all biomarkers, there is a gray zone of hs‐cTnT test results. In unselected patients hs‐cTnT concentrations <14 ng/L ruled out AMI with very high probability, and values >45 ng/L ruled in AMI with high probability, and in chest pain patients hs‐cTnT admission concentrations <5 ng/L ruled out AMI with very high probability, and concentrations >30 ng/L predicted AMI with high probability. Thorough assessment of AMI pretest probability by clinical history and symptoms will be increasingly important for correct clinical interpretation of hs‐cTnT test results and kinetics in the ED, and it is important to note, supported by our results, that an increased hs‐cTn concentration alone is not sufficient to make the diagnosis of AMI.
